# *Chlamydia Trachomatis* Infection in High-Risk Human Papillomavirus Based on Cervical Cytology Specimen

**DOI:** 10.31557/APJCP.2019.20.12.3843

**Published:** 2019

**Authors:** Soracha Sangpichai, Natcha Patarapadungkit, Chamsai Pientong, Tipaya Ekalaksananan, Surachat Chaiwiriyakul, Ratchaneekorn Thongbor, Phannatorn Sirivech, Porntip Jangsiriwitayakorn, Tippawan Triamwittayanon

**Affiliations:** 1 *Department of Pathology, *; 2 *Department of Microbiology, Faculty of Medicine, Khon Kaen University, Khon Kaen, Thailand. *

**Keywords:** Chlamydia trachomatis, human papillomavirus, cytology

## Abstract

**Objective:**

High-risk human papillomavirus (HR HPV) was associated with the development of cervical cancer. Asymptomatic Chlamydia trachomatis (*C. trachomatis*) infection is the most common bacterial, sexually-transmitted infection. This study aimed to investigate the association of *C. trachomatis* in positive HR HPV and the cytological results from liquid-based cytology (LBC).

**Methods:**

150 residual LBC specimens were collected; all of which had undergone cytology and HPV testing by Cobas. The samples were established as *C. trachomatis* using real-time PCR (RT-PCR) with Cryptic F/Cryptic R primers.

**Results:**

Of 150 positive HPV findings, the most common (72.7%, 109/150) were the 12 other HR HPVs (viz., 31, 33, 35, 39, 45, 51, 52, 56, 58, 59, 66, and 68). The cervical cytology of those positive HR HPVs were mostly negative (70.0%, 105/150). The *C. trachomatis* infections in positive HR HPV were 16% (24/150) HPV. The analysis of the abnormal cytology revealed that 41.6% had *C. trachomatis* co-infection (*C. trachomatis* and HPV infection) viz., LSIL (20.8%), HSIL (12.5%), and ASC-US (8.3%). A comparison with positive HPV without *C. trachomatis* co-infection revealed that the highest prevalence was for LSIL, ASC-US, and HSIL (11.1%, 10.3%, and 6.4%, respectively). There was no difference between the abnormalities and negative cervical cytology with negative and positive *C. trachomatis* co-infection in HR HPV positive (p = 0.174).

**Conclusion:**

*C. trachomatis* infection was not significantly associated HR-HPV and abnormal cytology. This study confirms the increasing rate of *C. trachomatis* infection in asymptomatic women so routine screening for these infections has been suggested to (a) prevent complications such as the chronic pelvic pain associated with prolong infection and (b) reduce sexual transmission of the infection.

## Introduction

Human papillomavirus (HPV) infection in women are usually asymptomatic and most clear spontaneously within 1–2 years (Jenkins, 2007). Persistent infection with various high-risk types of HPV (HR HPV) often progresses to invasive cancer. HPV DNA can be detected in more than 99% of cervical cancer, of which 70% are caused by HR HPV16 and 18 (Douglas, 2016). HPV16 was the most common type found in cervical carcinoma in all regions. The other associated risk factors for developing cervical cancer are first coitus age, multiple sexual partners, cigarette smoking, sexually-transmitted disease (STD), immunosuppression, oral contraceptive use, and co-infection with other sexually-transmitted infection (STI) (Jhingran et al., 2014). 

Chlamydia trachomatis (*C. trachomatis*) is the most common bacterial STI worldwide with more than 1.4 million infections in the United States alone (Geisler, 2016). *C. trachomatis* affects 7.8% of women with symptomatic lower genital tract infection as detected by cyclohexamide-treated McCoy cells (Rugpao et al., 1993). Among asymptomatic Thai males, 7.4% were positive by PCR for *C. trachomatis* in urine (Jiamton et al., 2019). Persons, especially women, with asymptomatic chlamydial infection are at high risk of spreading the pathogens to their partners and themselves suffering serious reproductive morbidity (i.e., to the upper genital tract leading to salpingitis, pelvic inflammatory diseases (PID), ectopic pregnancy, and infertility). Men infected with *C. trachomatis *are often diagnosed as having non-gonococcal urethritis (NGU) and complications may cause epididymitis or orchitis in the upper ascending genital tract. 

Several studies have suggested that *C. trachomatis* is a co-factor with HPV in the development of cervical cancer (Silins et al., 2005; Jensen et al., 2014). *C. trachomatis* can moreover establish asymptomatic, persistent infections by several mechanisms, including antibiotic resistance, immune evasion, and apoptosis (Chumduri et al., 2013). In addition, *C. trachomatis* is like other intracellular pathogens that can cause substantial changes in gene expression and protein production in the host at the transcriptional, translational, and post-translational levels. Whether the interaction between HPV and *C. trachomatis* infection will result in development of cervical cancer remains unclear (Elwell et al., 2016). The current study aimed to investigate the prevalence of *C. trachomatis* in persons positive and at high-risk of HPV (HR HPV) infection based on liquid-based cytology.

## Material and Methods

*Specimen collection and preparation*

The study protocol was reviewed and approved by the Ethics Committee of Khon Kaen University, Thailand (HE 591453). We collected 150 residual, routine, positive HPV DNA specimens based on liquid-based cytology (LBC) in ThinPrep^®^ between November, 2016 and November, 2018 using the Cobas^®^ 4800 system. The data comprised the individual results for HPV 16 and 18 and the pooled results for the 12 other HR HPV genotypes (viz., 31, 33, 35, 39, 45, 51, 52, 56, 58, 59, 66, and 68) processed at the Cytology Unit, Department of Pathology, Faculty of Medicine, Khon Kaen University. The cytological results were categorized following the 2014 Bethesda System as (a) negative for an intraepithelial lesion or malignant (NILM), (b) atypical squamous cells of undetermined significance (ASCUS), (c) low-grade squamous intraepithelial lesion (LSIL), (d) atypical squamous cells that cannot be ruled out as high-grade squamous intraepithelial lesion (ASC-H), (e) high-grade squamous intraepithelial lesion (HSIL), or (f) squamous cell carcinoma (SCC) (Nayar and Wilbur, 2015).

*DNA extraction*

The scraped cervical cells were washed twice with phosphate buffer saline (PBS). The cells were lysed in 200 µl of lysis buffer (10 mM Tris HCl, 0.1 mM EDTA pH 7.5, 1% SDS and 0.5 M NaCl), to which 15 µl of proteinase K was added. This was then mixed by vortex until clear then incubated at 60°C for 30 min. The protein was precipitated by addition of protein precipitation buffer (5 M potassium acetate, 11.5 ml of glacial acetic acid and 28.5 ml of distill water, pH 5.5): the mixture was centrifuged at 13,500 rpm for 5 min at 4°C. The DNA was precipitated with an equal volume of isopropanol and collected by centrifugation at 13,500 rpm for 5 min at 25°C and washed with 70% ethanol. The DNA pellet was air-dried for 15-30 min then re-suspended in 40 µl of distilled water and stored at -20°C until used.

*C. trachomatis DNA detection*

The quality of extracted DNA was checked by amplification of a housekeeping gene (Glyceraldehyde 3-phosphate dehydrogenase; GAPDH). The extracted DNA was determined using GAPDH gene detection. *C. trachomatis* DNA was detected using SYBR real-time PCR (RT PCR) with Cryptic F/Cryptic R primers. The primers were applied for a Chlamydia cryptic plasmid investigation, which amplifies the 71 bp DNA fragment between the open reading frame 1 and 2 (Jaton et al., 2006). The real-time master mix PCR for the cryptic plasmid region (amplifying ABI 7500 fast, Applied Biosystems) contained 5.0 µl of SYBR (SsoAdvancedTM Universal SYBR®Green Supermix), 0.3 µl of each primer, 2.4 µl of distilled water, and 2 µl of DNA sample. Amplification was performed by a pre-warming step at 50°C for 2 min, followed by denaturing at 95°C for 10 sec, 45 cycles at 95°C for 15 sec, and 60°C for 1 min. 

*Statistical analysis*

The chi-square test was used to analyze the correlation between HPV and *C. trachomatis* DNA detection. A *p-value* < 0.05 was considered significant. All statistical tests were performed using STATA version 10. 

## Results

*Genotype of HPV by cobas® 4800 system*

**Figure 1:**
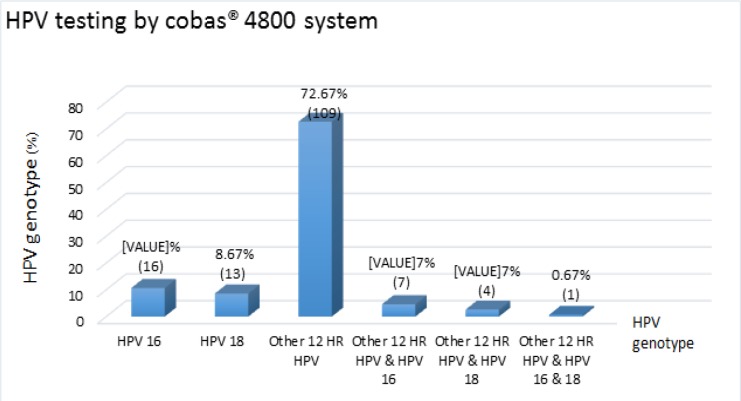
Frequency of 150 Positive HPV Genotype by cobas® 4800 System

The 150 positive HPV testing by Cobas^®^ 4800 system revealed 10.7% (16/150) HPV 16, 8.6% (13/150) HPV 18, 72.7% (109/150) 12 other HR HPV (31, 33, 35, 39, 45, 51, 52, 56, 58, 59, 66, and 68), 4.7% (7/150) 12 other HR HPV plus HPV 16, 2.7% (4/150) 12 other HR HPV plus HPV 16, and 0.7% (1/150) 12 other HR HPV plus HPV 16 and 18 (Figure 1).

*Cytological results in positive HR HPV*

**Figure 2:**
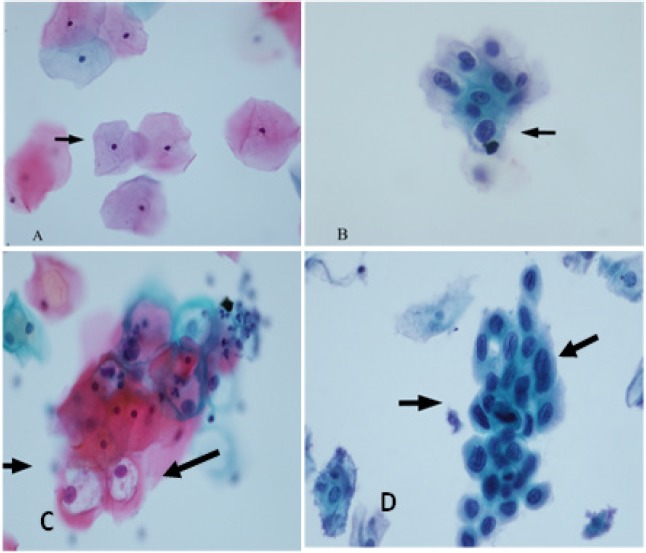
Cytological Samples Classified into Three Groups According to the 2001 Bethesda System. A, negative for intraepithelial lesion or malignancy within normal limits; B, atypical squamous cells of undetermined significance (ASCUS); C, low-grade squamous intraepithelial lesion (LSIL); D, high-grade squamous intraepithelial lesion(HSIL) by Papanicolaou stain (x400)

The cytological morphology has been reported as NILM, ASCUS, LSIL, and HSIL by the Cytology Unit (Figure 2). The 150 positive HPVs comprised 70.0% (105/150) negatives, 10.0% (15/150) ASC-US, 12.7% (19/150) LSIL, and 7.3% (11/150) HSIL. The negative cytologies found included 76.2% (80/105) of the 12 other HR HPVs, followed by 10.5% (11/105) of HPV 16 and 6.7% (7/105) of HPV 18. In addition, there were the 12 other HRs, HPV 16, and/or HPV. In the abnormal cytological group, the 12 other HR HPVs included ASC-US, LSIL, and HSIL (Table 1).

*Chlamydia cryptic plasmid detection*

**Figure 3:**
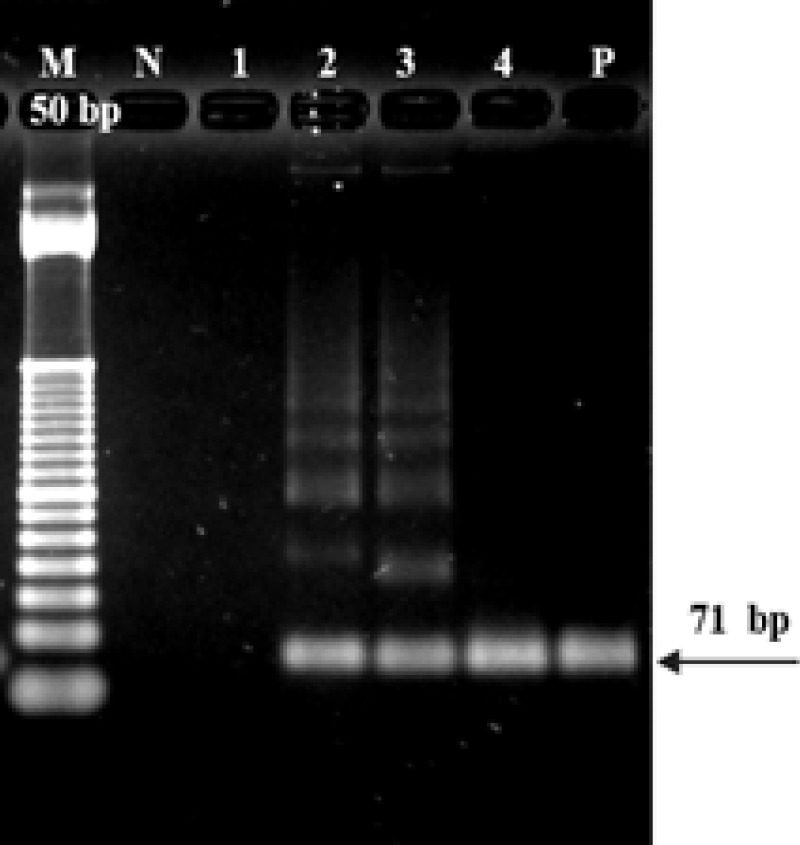
PCR Products of *C. trachomatis* DNA by Cryptic-F/Cryptic-R primers were separated on 2% agarose gel electrophoresis; M, DNA markers; P, positive control, and 1-4 = samples

The positive HPV testing detected the chlamydia cryptic plasmid DNA by real-time PCR using the Cryptic-F/Cryptic-R primers. DNA fragments were located between ORF1 and ORF2 of *C. trachomatis*. The melting temperature (Tm) of the cryptic plasmid was determined using Cryptic-F/Cryptic-R real-time PCR at between 79 and 83°C. The optimal temperature per the melting curve was Tm 81 ºC. The 71 bp of the PCR products were tested using 2% agarose gel electrophoresis stained with ethidium bromide (Figure 3). The *C. trachomatis* infections found included 16% (24/150) HPV of which the 12 other HR HPV genotypes were the most frequent (66.7%) (16/24) positive for *C. trachomatis* while HPV 18 was not found (Table 2).

*Association between cytological results and C. trachomatis infection*

We observed an association between abnormal cervical cytology and *C. trachomatis* in the positive HR HPV. The analysis of 41.6% (10/24) of abnormal cytology (ASC-US, LSIL and HSIL) revealed the highest prevalence of *C. trachomatis* co-infection in LSIL, followed by HSIL, and ASC-US (20.8%, 12.5%, and 8.3%, respectively). The comparison to positive HPV without *C. trachomatis* co-infection revealed that the highest prevalence was for LSIL, followed by ASC-US, and HSIL (11.1%, 10.3%, and 6.4%, respectively). The 72.2% (91/126) negative cervical cytology without *C. trachomatis* co-infection was higher than the 58.3% (14/24) *C. trachomatis* co-infection. There was no significant difference between the abnormalities (ASC-US, LSIL, and HSIL) and negative cervical cytology results of *C. trachomatis* co-infection in positive HR HPV cases (p = 0.174). Interestingly, the highest OR was for HSIL, 2.62 (0.4-12.8), followed by LSIL 2.32 (0.6-8.2) and ASCUS 1.00 (0.1-5.2) (Table 3). 

**Table 1:** Comparison of the Frequency Distributions of Cytological Diagnosis and HPV Typing

Positive HPV typing by cobas® 4800 system	Cytological diagnosis (n=150)			
	Negative (%)	ASC-US (%)	LSIL (%)	HSIL (%)
Other 12 HR HPV	80 (76.19)	12 (80)	11 (57.89)	6 (54.55)
HPV 16	11 (10.47)	2 (13.33)	2 (10.53)	1 (9.09)
HPV 18	7 (6.67)	1 (6.67)	3 (15.79)	2 (18.18)
Other 12 HR HPV, HPV 16	4 (3.81)	0	2 (10.53)	1 (9.09)
Other 12 HR HPV, HPV 18	2 (1.9)	0	1 (5.2)	1 (9.09)
Other 12 HR HPV, HPV 16, 18	1 (0.95)	0	0	0
Total (n=150)	105 (70)	15 (10)	19 (12.67)	11 (7.33)

**Table 2:** Frequency of *C. trachomatis* Detection by SYBR Green Real Time PCR of Each HPV Genotype by cobas® 4800 System

150 HPV genotype	24 (16.0%) Positive C. trachomatis
HPV 16	3 (12.5%)
HPV 18	0 ( 0%)
Other 12 HR HPV	16 (66.6%)
Other 12 HR HPV & HPV 16	3 (12.5%)
Other 12 HR HPV & HPV 18	1 (4.2%)
Other 12 HR HPV & HPV 16 & 18	1 (4.2%)

**Table 3:** Association between Cytology and *C. trachomatis* Infection in Positive HR HPV

Cytology diagnosis	Positive HPV (n=150)		OR (95%CI)	Total	p-value
	Positive C. trachomatis (n=24)	Negative C. trachomatis (n=126)			
Negative	14 (58.3%)	91 (72.2%)		105	0.174
ASC-US	2 (8.3%)	13 (10.3%)	1.00 (0.1 – 5.2)	15	
LSIL	5 (20.8%)	14 (11.1%)	2.32 (0.6 – 8.2)	19	
HSIL	3 (12.5%)	8 (6.4%)	2.62 (0.4 – 12.8)	11	
Total	24	126		150	

## Discussion

Sexually-transmitted viral infections of HPV in the genital tract are common. HPV infection may have transient symptoms, and spontaneous clearance occurs in 80% of cases as the virus is flushed by the host immune system without any cell changes. In approximately 20% of women, HPV infection may be persistent and later evolve into cervical cancer in up to 10% of cases (Abreu et al., 2012; Wohlmeister et al., 2016). Actually, the most important viral factor is the type of HPV. HR HPV genotypes may infect the epithelium persistently, inducing progression and contributing to carcinogenesis. Studies have shown that the most common HR HPV detected in carcinoma cases are HPV 16, 18, 31, 33, 39, and 45 (Muñoz 2000; Kraus et al., 2006; Parkin et al., 2008; Wohlmeister et al., 2016a). Kaliff et al., ( 2018) carried out a study and found that HPV 16 was the most prevalent genotype, followed by HPV 18: the two are the main genotypes detected in squamous cell carcinoma (Oliveira-Silva et al., 2011; Wohlmeister et al., 2016). The 12 other high-risk HPV genotypes (viz., HPV 31, 33, 35, 39, 45, 51, 52, 56, 58, 59, 66, and 68) were highly prevalent in the present study; more in fact than HPV 16 or HPV 18. 

The prevalence of *C. trachomatis* among asymptomatic women positive for HR HPV in the current study was 16%. The prevalence of genital chlamydia infection in the current study was higher than in previous studies. Thongkrajai et al., (1999) reported a respective prevalence of *C. trachomatis* using the ELISA method of 6.8%, 5.2%, and 6.7% in rural northeast Thai women attending antenatal, postpartum, and family planning clinics. The prevalence of *C. trachomatis* was investigated using multiplex PCR among students from the northern region of Thailand: the prevalence was between 3.2% to 7.5% (Whitehead et al., 2008). In Hanoi, Vietnam, the prevalence by ELISA technique of lower genital tract *C. trachomatis* infection was 3.8% in asymptomatic women attending maternal and child health and family planning clinics (Anh et al., 2003). By comparison, the prevalence of *C. trachomatis* infection in the United States was 1.7% (Torrone et al., 2014). In Iran, nested polymerase chain reaction (PCR) used to test the residual fluids of Pap smears: 12.5% were positive for *C. trachomatis* infection (Javanmard et al., 2018). Differences in study populations and methodologies used for *C. trachomatis* detection yield a wide variation in prevalence rates. The prevalence also varies by age and race/ethnicity. Common co-infections are often asymptomatic, so healthcare providers should routinely screen sexually active women and provide prompt treatment for infected persons (Torrone et al., 2014). 

Safaeian et al., (2010) *C. trachomatis* infection affects HPV persistence and progression to cervical pre-malignancy by a causal disruption of the epithelial tissue. De Paula et al., (2007) reported that although a significant association was found for HPV infection and the precursor lesions of cervical cancer, it was not possible to establish a significant association between these lesions and *C. trachomatis* co-infection. In the current study, no significance was found between the abnormalities and negative cervical cytology with *C. trachomatis* in HR HPV positive co-infection (p = 0.174). The highest OR presented in HSIL (2.62 [0.4-12.8]) followed by the LSIL and ASCUS groups (2.32 [0.6-8.2] and 1.00 [0.1-5.2]) (Table 3). The small number of cases of abnormal cytology in HR HPV make it difficult to confirm whether there are significant differences among the different types of *C. trachomatis* infections.

In conclusion, there were no significantly associated risk factors between *C. trachomatis* infection and HR-HPV infection. The abnormalities of cervical premalignancy are thus not likely associated with *C. trachomatis* in HR HPV co-infection. Results from the current study confirmed the observation of increasing *C. trachomatis* infection among asymptomatic women. Routine screening and appropriate treatment for these infections should be performed to prevent complications such as (a) chronic pelvic pain in women, (b) prolonged *C. trachomatis* infection, and (c) reduced spreading to sexual partners. 
